# Genome-Resolved Metagenomic Insights into Massive Seasonal Ammonia-Oxidizing Archaea Blooms in San Francisco Bay

**DOI:** 10.1128/msystems.01270-21

**Published:** 2022-01-25

**Authors:** Anna N. Rasmussen, Christopher A. Francis

**Affiliations:** a Department of Earth System Science, Stanford University, Stanford, California, USA; University of Illinois at Chicago

**Keywords:** estuary, ammonia-oxidizing archaea, *Thaumarchaeota*, nitrification, bloom, pelagic, metagenome-assembled genome

## Abstract

Ammonia-oxidizing archaea (AOA) are key for the transformation of ammonia to oxidized forms of nitrogen in aquatic environments around the globe, including nutrient-rich coastal and estuarine waters such as San Francisco Bay (SFB). Using metagenomics and 16S rRNA gene amplicon libraries, we found that AOA are more abundant than ammonia-oxidizing bacteria (AOB) and nitrite-oxidizing bacteria (NOB), except in the freshwater stations in SFB. In South SFB, we observed recurrent AOA blooms of “*Candidatus* Nitrosomarinus catalina” SPOT01-like organisms, which account for over 20% of 16S rRNA gene amplicons in both surface and bottom waters and co-occur with weeks of high nitrite concentrations (>10 μM) in the oxic water column. We observed pronounced nitrite peaks occurring in the autumn for 7 of the last 9 years (2012 to 2020), suggesting that seasonal AOA blooms are common in South SFB. We recovered two high-quality AOA metagenome-assembled genomes (MAGs), including a *Nitrosomarinus*-like genome from the South SFB bloom and another *Nitrosopumilus* genome originating from Suisun Bay in North SFB. Both MAGs cluster with genomes from other estuarine/coastal sites. Analysis of *Nitrosomarinus*-like genomes show that they are streamlined, with low GC content and high coding density, and harbor urease genes. Our findings support the unique niche of *Nitrosomarinus*-like organisms which dominate coastal/estuarine waters and provide insights into recurring AOA blooms in SFB.

**IMPORTANCE** Ammonia-oxidizing archaea (AOA) carry out key transformations of ammonia in estuarine systems such as San Francisco Bay (SFB)—the largest estuary on the west coast of North America—and play a significant role in both local and global nitrogen cycling. Using metagenomics and 16S rRNA gene amplicon libraries, we document a massive, recurrent AOA bloom in South SFB that co-occurs with months of high nitrite concentrations in the oxic water column. Our study is the first to generate metagenome-assembled genomes (MAGs) from SFB, and through this process we recovered two high-quality AOA MAGs, one of which originated from bloom samples. These AOA MAGs yield new insight into the *Nitrosopumilus* and *Nitrosomarinus*-like lineages and their potential niches in coastal and estuarine systems. *Nitrosomarinus*-like AOA are abundant in coastal regions around the globe, and we highlight the common occurrence of urease genes, low GC content, and range of salinity tolerances within this lineage.

## INTRODUCTION

Nitrogen is an essential element for all life on Earth, but due to human activities, it has also become a major pollutant in estuarine and coastal systems (1). San Francisco Bay (SFB) is the largest estuary on the west coast of North America and is highly N polluted from agricultural and urban runoff, as well as large inputs from wastewater treatment plants (WWTPs). SFB consists of two distinct but connected arms, generally referred to as North and South SFB, and 5 major subembayments, including Suisun Bay, San Pablo Bay, Central Bay, South Bay, and Lower South Bay, each with different sources and forms of dissolved inorganic nitrogen (DIN) inputs (2). Wastewater treatment plants discharge high levels of ammonia into SFB, especially in South Bay (2, 3). This excess ammonia has many potential fates once it reaches SFB waters, including consumption/assimilation by microorganisms as well as oxidation to nitrite and ultimately nitrate via the chemoautotrophic process of nitrification. Nitrification generally occurs in two steps, ammonia and then nitrite oxidation. The organisms responsible for the first step, aerobic ammonia oxidation, include ammonia-oxidizing archaea (AOA) and ammonia-oxidizing bacteria (AOB), while the second step is carried out by nitrite-oxidizing bacteria (NOB). Comammox bacteria are capable of carrying out both ammonia and nitrite oxidation.

Understanding the microorganisms responsible for the transformation of ammonia to nitrate in the SFB water column has implications for primary production and potential N loss. High ammonia concentrations can lead to eutrophication in estuaries (4), though currently, phytoplankton dynamics in SFB are strongly influenced by light limitation (5–7) and grazing (8, 9), leading to a high-nutrient, low-chlorophyll state in parts of SFB. However, the form of DIN could also be one of several factors influencing phytoplankton dynamics and composition in SFB (10–16), potentially linking rates of nitrification to phytoplankton community composition. Nitrate produced by nitrification can also be denitrified in suboxic/anoxic sediments of the bay (17, 18) and lost from the system as N gases (e.g., N_2_ and N_2_O). While nitrification rate measurements are limited for SFB, studies have found rates to vary seasonally, geographically, and by water column depth (19, 20). For example, rates are highest in ammonia-rich waters near the Sacramento regional water treatment plant (20), tend to be higher in bottom waters than in surface waters (19), and are linked to suspended particulate matter (SPM) dynamics (19).

Despite the importance of nitrification to N cycling in SFB, only a few studies have investigated the ecology of benthic nitrifiers in SFB (21, 22), and nitrifiers in the water column remain virtually unstudied. Several AOA have been successfully enriched, isolated, and sequenced from North SFB sediments, yielding insights into low-salinity and brackish sediment organisms, including the genetic capacity for mechanosensitive channels for osmotic regulation and motility (23–26). Clone library and qPCR-based studies based on *amoA* genes (encoding ammonia monooxygenase subunit A) have had mixed results, with either AOB or AOA being numerically dominant in low-salinity sediments of North SFB (21, 22) and AOB being more abundant in higher-salinity sediments of the bay (21). Both AOB and AOA have been shown to be more abundant than the other depending on the estuary (27–34), though several studies have found AOA to be dominant in nutrient-enriched estuarine waters (29, 35). Of particular relevance to this study, seasonal AOA blooms and the decoupling of ammonia and nitrite oxidation have been observed in estuarine waters around the globe (36, 37); however, such blooms have yet to be examined using genome-resolved metagenomics. In this study, we used genomic binning and analysis approaches to assess nitrifier populations and generate the first metagenome-assembled genomes (MAGs) from SFB.

MAGs have greatly expanded our ability to examine the metabolic capacity of organisms from a wide variety of environments. For example, MAGs have already increased our understanding of different niches within the AOA (38, 39) and allow us to study organisms that have yet to be enriched or isolated in the laboratory and/or that may have been missed by previous primer-based studies. The discovery of an increasing number of AOA MAGs allows us to compare a large number of otherwise undiscoverable genomes to gain insight into organisms from the estuarine environment.

A previous 16S rRNA gene amplicon-based study of the SFB water column revealed a high abundance of *Nitrosopumilus*-like organisms in South SFB along with high nitrite concentrations (40). Here, we delved much deeper into this initial finding by including metagenomic sequencing, as well as additional 16S rRNA gene amplicon libraries analyzed via amplicon sequence variants (ASVs) versus a 97% operational taxonomic unit (OTU)-based approach. We assessed both surface and bottom water amplicon libraries from South Bay. We recovered two high-quality MAGs (>97% complete and <1% contamination), including a “*Candidatus* Nitrosomarinus catalina” SPOT01-like population genome and a *Nitrosopumilus* lineage. We compared the genomic characteristics of our MAGs to AOA genomes from other estuarine and coastal environments and leveraged our 16S rRNA gene time series to better understand the spatial and temporal distribution of temporally abundant AOA in SFB.

## RESULTS AND DISCUSSION

We recovered two high-quality AOA MAGs from SFB after dereplicating 21 *Nitrosopumilus*-like MAGs (>70% complete, <5% contamination) (Table S1) at 98% average nucleotide identity (ANI) (Fig. S1). The two representative MAGs are designated SFB_27D_13Oct24_05_ms_bin_1 (97.6% complete, 0.0% contamination) and SFB_3D_13Oct25_100_mh_bin_18 (100.0% complete, 0.97% contamination), referred to here as SFB_27_05_bin1 and SFB_3_bin18. Both of these MAGs have 16S rRNA and *amoA* genes. Additionally, a different MAG, SFB_27D_13Oct24_03_ms_bin1, was used in place of SFB_27_05_bin1 in the concatenated ribosomal tree because several ribosomal genes are missing from SFB_27_05_bin1. According to the GTDB-tk classification, SFB_27_05_bin1 is classified as “*Nitrosopumilus catalinensis*” with an ANI of 99.23% to the “*Ca.* Nitrosomarinus catalina” SPOT01 (GCF_002156965.1), which was enriched from the water column in the San Pedro Channel near Santa Catalina Island off the coast of Los Angeles, CA, USA (41). SFB_3_bin18 is only classified to the genus level of *Nitrosopumilus* and most closely related to “*Nitrosopumilus* sp006740685,” with an ANI of 85.26% to *Nitrosopumilus* sp. strain SW (GCF_006740685.1), which was isolated from coastal surface waters (20 m) of the Yellow Sea on the west coast of the Korean peninsula (42). This MAG likely represents a new species based on the GTDB-tk classification. Information on the quality of all AOA MAGs generated in this study is available in Table S1. No other nitrifier MAGs were recovered.

10.1128/mSystems.01270-21.1 FIG S1 ANI calculation for genome clusters identified using dRep for MAGs generated from SFB. (A) SCM1-like MAGs and (B) SPOT01-like MAGs. Download FIG S1, EPS file, 0.1 MB. Copyright © 2022 Rasmussen and Francis. 2022 Rasmussen and Francis. https://creativecommons.org/licenses/by/4.0/ This content is distributed under the terms of the Creative Commons Attribution 4.0 International license.

10.1128/mSystems.01270-21.8 TABLE S1 Ammonia-oxidizing archaea bin metadata. Download Table S1, PDF file, 0.04 MB. Copyright © 2022 Rasmussen and Francis. 2022 Rasmussen and Francis. https://creativecommons.org/licenses/by/4.0/ This content is distributed under the terms of the Creative Commons Attribution 4.0 International license.

### South Bay AOA MAG and ASV abundance correlated with high nitrite concentrations.

**FIG 1:**
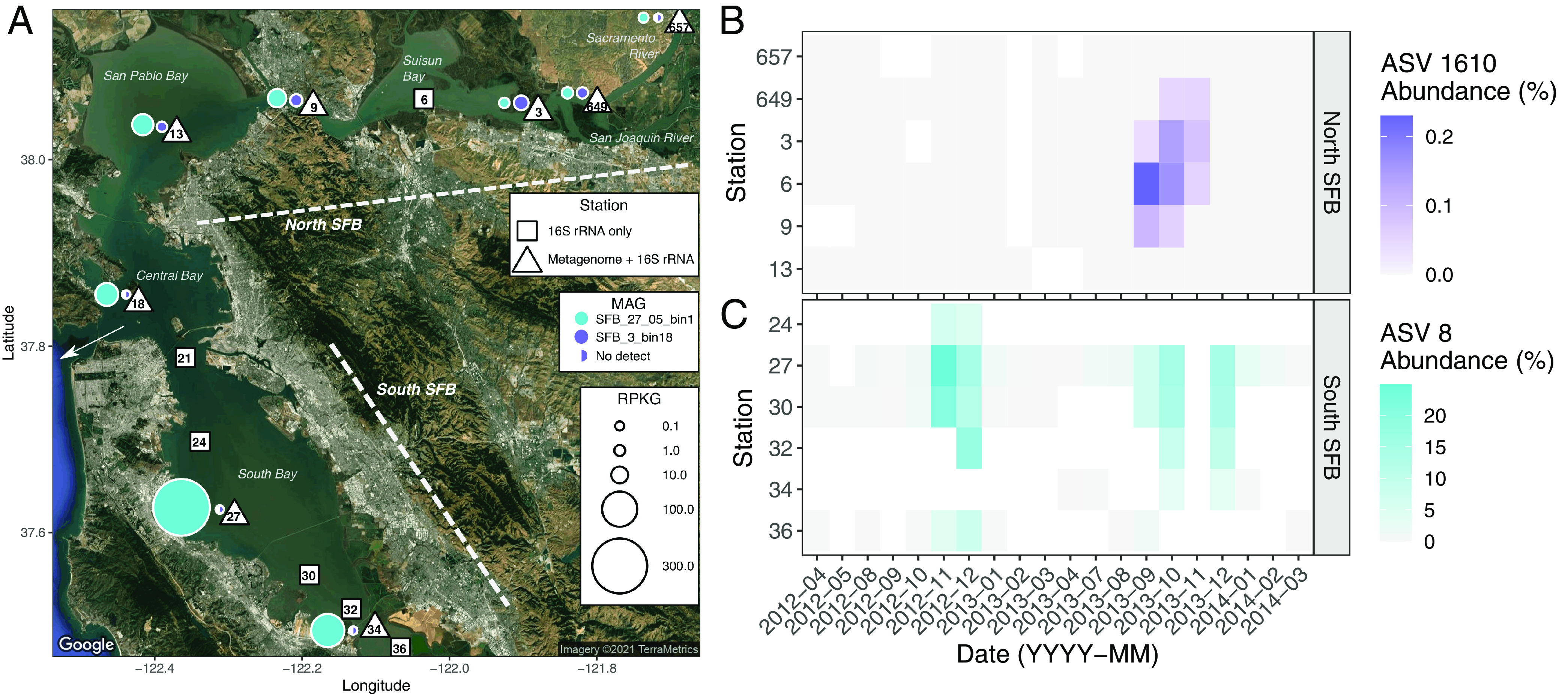
(A) Metagenome reads recruited to two high-quality representative AOA MAGs from SFB. White triangles or squares indicate station location for samples with 16S rRNA gene amplicons (squares) plus metagenomes (triangles). Circle size represents reads per kilobase of genome per gigabase of metagenome (RPKG) recruited to the respective MAG, which is indicated by circle color. SFB_3_bin18 was not detected in 4 samples, represented by half-filled circles. The white arrow indicates where SFB connects to the Pacific Ocean. (B and C) Tile plots indicate the relative abundance in 16S rRNA amplicon libraries of (B) ASV 1610 and (C) ASV 8, which correspond to the 16S rRNA gene of MAGs SFB_3_bin18 and SFB_27_05_bin1, respectively, over a 2-year monthly time series (April 2012 to March 2014). Panel B includes only bottom water samples, while panel C includes the mean abundance of surface and bottom water samples.

**FIG 2:**
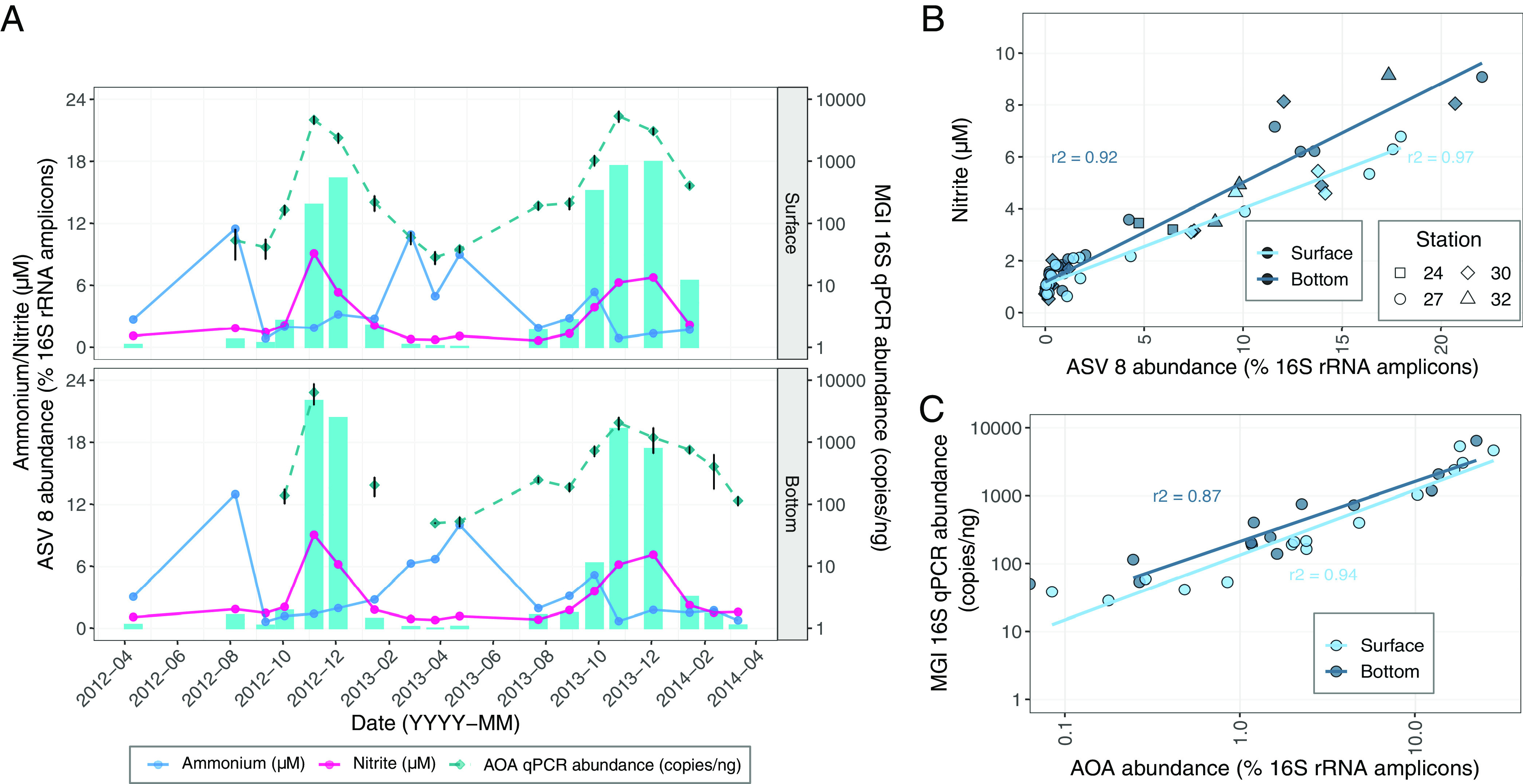
Abundance of ASV 8 in 16S rRNA gene amplicon libraries corresponds to temporal patterns in nitrite and ammonia concentrations in South Bay in both surface water (2 m) and bottom water (1 m above the estuary floor). (A) ASV 8 abundance (bars) at station 27 along with nitrite and ammonium concentrations and MGI *Thaumarchaeota* 16S rRNA gene qPCR abundance over the course of a 2-year approximately monthly time series (April 2012 to March 2014). qPCR values are on a log scale. (B) Nitrite concentrations versus ASV 8 abundance in several South Bay stations, including 24, 27, 30, and 32, is highly correlated in both surface (*r*^2^ = 0.97) and bottom (*r*^2^ = 0.92) waters. (C) AOA abundance measured via qPCR versus the relative abundance of AOA in 16S rRNA gene amplicon libraries is correlated in surface (*r*^2^ = 0.94) and bottom (*r*^2^ = 0.87) waters. Both axes are on a log scale.

We aligned the 16S rRNA V4-V5 region from our two representative AOA MAGs with ASVs from our 16S rRNA gene amplicon library to assess spatiotemporal distribution of these representative organisms and gain further insight into their environmental distribution. Both metagenomic and 16S rRNA gene ASV analysis revealed a highly abundant “*Ca.* Nitrosomarinus catalina”-like lineage. SFB_27_05_bin1 recruits 6.0% of metagenomic reads from station 27 and has a maximum of 329 RPKG (reads recruited per kilobase of MAG per gigabase of metagenome) at this station (Fig. 1). ASV 8 (the 8th most abundant ASV) in our 16S rRNA gene amplicon data set is identical to the 16S V4-V5 region of SFB_27_05_bin1, indicating that this MAG represents the same population of AOA observed in the amplicon libraries (Table S2). We observed a pronounced recurring bloom in South Bay of ASV 8 in the fall of 2012 and 2013 in both bottom and surface waters (Fig. 1 and 2), when salinities are generally over 30 practical salinity units (PSU) and temperatures are between 16 and 20°C (Fig. S2A). The abundance of ASV 8 is strongly correlated with nitrite concentrations in South Bay in both surface (*r^2^* = 0.97, *P* < 0.001) and bottom (*r^2^* = 0.92, *P* < 0.001) waters (Fig. 2). Ammonia concentrations are not significantly correlated with ASV 8 abundance (*r^2^* = 0.06, *P* > 0.05). The abundance of SFB_27_05_bin1 is also correlated with nitrite concentrations (*r^2^* = 0.94, *P* < 0.001). The abundance of AOA (measured via quantitative PCR [qPCR] analysis of marine group I [MGI] *Thaumarchaeota* 16S rRNA genes) was also correlated with nitrite concentrations (*r^2^* = 0.88, *P* < 0.001) and with the relative abundance of AOA in 16S rRNA amplicon data (*r^2^* = 0.78, *P* < 0.001). Based on qPCR data, AOA abundance increased 2 orders of magnitude from nonbloom to bloom samples (Fig. 2). Nutrient data collected by the U.S. Geological Survey (USGS) show that high surface nitrite concentrations in South Bay have recurred in 7 of the last 9 years (2012 through 2020), generally peaking in October through December and reaching almost 12 μM in some years (Fig. S2B). The high AOA abundance in both years of 16S rRNA gene amplicon data and qPCR data (2012 and 2013) and strong correlation to nitrite concentrations suggest that AOA blooms are a recurrent feature in South Bay during the midfall to late fall, leading to this semiregular nitrite accumulation (Fig. 2).

10.1128/mSystems.01270-21.2 FIG S2 (A) Environmental data for stations 3, 6, and 27 over the course of the 2-year time series in surface (left) and bottom (right) waters. SPM is transformed by the natural log. (B) Maximum monthly surface nitrite concentrations measured by USGS from 2012 through 2020 for Suisun Bay (stations 2 through 11; top) and South Bay (stations 27 through 32; bottom). (C) Light extinction coefficient (measured as the slope of the regression line between the natural log of photosynthetically active radiation [PAR] and water depth) versus SPM concentrations is correlated (*r*^2^ = 0.82) at station 27. Samples from the AOA bloom are indicated by dark orange triangles. Download FIG S2, EPS file, 0.7 MB. Copyright © 2022 Rasmussen and Francis. 2022 Rasmussen and Francis. https://creativecommons.org/licenses/by/4.0/ This content is distributed under the terms of the Creative Commons Attribution 4.0 International license.

10.1128/mSystems.01270-21.9 TABLE S2 Similarity of 16S rRNA V4-V5 regions from representative AOA MAGs and top 20 *Thaumarchaeota* ASVs in 16S rRNA gene amplicons. Download Table S2, PDF file, 0.04 MB. Copyright © 2022 Rasmussen and Francis. 2022 Rasmussen and Francis. https://creativecommons.org/licenses/by/4.0/ This content is distributed under the terms of the Creative Commons Attribution 4.0 International license.

During this time of high AOA abundance, we found nitrite-oxidizing bacteria (NOB) to generally be 2 orders of magnitude less abundant than AOA, reaching a maximum relative abundance of 0.7% in 16S rRNA gene amplicon libraries in South SFB in December 2013 (after the onset of the AOA bloom in October 2013 [Fig. S3]). NOB detected in SFB belonged to the genera *Nitrospira*, *Nitrospina*, and LS-NOB (*Nitrospinae* clade 2 [43]). Despite high dissolved inorganic nitrogen (DIN) concentrations in SFB waters, no NOB from the phylum *Proteobacteria* were detected (e.g., “*Candidatus* Nitrotoga,” *Nitrolancea*, *Nitrobacter*, or *Nitrococcus*). *Nitrospira* organisms were found throughout SFB, from low-salinity sites in the North SFB to near-marine-salinity sites in South SFB, while *Nitrospina* organisms were found in higher-salinity sites and had the highest abundances in South SFB. LS-NOB are found around the globe in temperate and tropical ocean waters and can be numerically dominant; within SFB, LS-NOB were the dominant NOB at the most marine-influenced site, station 18 (Fig. S3). Ammonia-oxidizing bacteria (AOB) consisted solely of *Nitrosomonas* and were of similarly low abundance to NOB, with a maximum of 0.2% in 16S rRNA gene amplicon libraries. In general, NOB and AOB had much lower abundance than AOA and had abundances only comparable to those of AOA in freshwater stations where all three nitrifier groups were of low relative abundance (Fig. S3).

10.1128/mSystems.01270-21.3 FIG S3 Nitrifier abundances (excluding ASV 8, which represents the AOA bloom lineage) in 16S rRNA gene amplicon libraries from select seasonally representative cruises over the 2-year time series. The scale is the same in each panel; however, the panel height varies. Download FIG S3, EPS file, 0.3 MB. Copyright © 2022 Rasmussen and Francis. 2022 Rasmussen and Francis. https://creativecommons.org/licenses/by/4.0/ This content is distributed under the terms of the Creative Commons Attribution 4.0 International license.

High nitrite concentrations due to the decoupling of ammonia and nitrite oxidation in the water column have been reported in many other estuaries and coastal sites (36, 37, 44–47). Although low oxygen concentrations can contribute to the decoupling of ammonia and nitrite oxidation in natural and engineered systems (46–51), a meta-analysis of 29 estuaries and lagoons found that transient nitrite accumulation was driven primarily by higher temperatures rather than hypoxia (36). Schaefer and Hollibaugh (36) found peak nitrite occurring in late summer into early fall, driven by warmer temperatures between 20 and 30°C. AOA blooms have been observed in estuaries and coastal bays in the late summer (37), fall (40, 52), and winter (53) and after wind events that cause vertical mixing, bringing oxygenated waters into deeper, ammonium-rich waters (46). In another example, in two bays of the Yellow Sea, high *Nitrosopumilus*-like OTU abundance was concomitant with euryarchaeal blooms in October, suggesting that euryarchaeal heterotrophy could provide ammonia for ammonia oxidation (52). During this bloom in the Yellow Sea, *Nitrosopumilus*-like OTUs reached over 30% relative abundance and were correlated with nitrite concentrations, which reached 4 μM (52).

The decoupling of ammonia and nitrite oxidation in SFB does not obviously follow patterns observed in other estuaries. For example, in seasonally stratified systems, such as the Chesapeake Bay, transient wind events have led to nitrite accumulation when ammonia-rich and oxygen-poor waters are mixed, allowing significant ammonia but not nitrite oxidation (46). In contrast, SFB waters (especially in the channel where our samples were taken) are oxic and well mixed (Fig. S2A), suggesting that dissolved oxygen (DO) is not likely to be an explanatory factor. The AOA bloom in South SFB also occurs in fall, after peak summer temperatures have occurred (Fig. S2A), in sharp contrast to what has been observed in a global analysis of nitrite accumulation in estuaries (36). It is worth highlighting that “*Ca.* Nitrosomarinus catalina” SPOT01 has a lower optimal growth rate and outperforms other cultured marine *Thaumarchaeota* strains at temperatures of <20°C (41). Thus, the decoupling of ammonia and nitrite oxidation in SFB is not likely caused by low DO or warm temperatures; however, cooler temperatures could potentially lead to differences between AOA and NOB activity.

One potentially noteworthy environmental factor in SFB is the relatively low turbidity during the bloom period (Fig. S2A), which generally leads to greater light penetration (Fig. S2C). The relative photosensitivity of AOA versus AOB versus NOB varies and has been documented in the ocean, estuaries, and WWTPs (54–59). Although cultivated AOA and NOB can be more sensitive than AOB to photoinhibition (55, 59), some oceanic AOA are still active under high irradiance (60). Ammonia oxidation has also been shown to be less sensitive to light than nitrite oxidation in the sunlit upper ocean (where AOA predominate over AOB), contributing to the formation of primary nitrite maxima (61–63). Similarly, it is indeed possible that NOB in SFB are more photosensitive than the AOA lineage that blooms, leading to their decoupled abundance and activity. At station 27, the relative abundance of both *Nitrospina* and *Nitrospira* is positively correlated with the natural log of SPM concentrations (*r^2^* = 0.59 and *r^2^* = 0.70, respectively; *P* < 0.001), which could be related to photoinhibition or particle association. Overall, the potential role of differential light inhibition during the AOA bloom is intriguing and warrants further physiological studies of relevant AOA and NOB. Finally, we also did not observe a concomitant euryarchaeal bloom with the AOA bloom, as observed in the Yellow Sea (52), suggesting that enhanced euryarchaeal heterotrophy is not a source of additional ammonia for AOA. A co-occurrence network identified 11 ASVs with associations with bloom-forming ASV 8, none of which belonged to the *Euryarchaea* or NOB. Of the 11 ASVs, we highlight three ASVs with peak abundances occurring during the AOA bloom belonging to *Bacteroidota*, *Dadabacteria*, and the NS11-12 marine group (*Sphingobacteriales*) (Fig. S4). The associations of these ASVs could indicate possible positive interactions during the bloom or similar ecophysiological responses to seasonal conditions.

10.1128/mSystems.01270-21.4 FIG S4 Relative abundance of ASVs in 16S rRNA amplicon data with associations in co-occurrence networks made using SPIEC-EASI to (A) ASV 8 at station 27 (bottom waters only) and (B) ASV1610 at station 6. Figures include ASVs with both similar and opposing seasonal trends to the AOA ASVs of interest. Download FIG S4, EPS file, 0.1 MB. Copyright © 2022 Rasmussen and Francis. 2022 Rasmussen and Francis. https://creativecommons.org/licenses/by/4.0/ This content is distributed under the terms of the Creative Commons Attribution 4.0 International license.

Given that SPOT01-like organisms have been found to be dominant not only during the AOA bloom in SFB but in estuarine and coastal sites across the globe in 16S rRNA, *amoA*, and metagenomic studies (31, 38, 41, 64, 65), key environmental parameters in addition to cooler temperatures, as mentioned above, likely influence when and where SPOT01-like AOA thrive. Salinity could also be an important influence on the dominance of SPOT01-like organisms. In the Jiulong River estuary (JRE), a SPOT01-like *amoA* OTU is dominant at salinities ranging from 15.8 to 27.0 PSU (65); in the Pearl River estuary (PRE), SPOT01-like MAGs are also most abundant in saltier stations (64); and SPOT01 dominates at the San Pedro Ocean time series (SPOT) off the coast of California in September and October (41). These findings indicate that SPOT01-like organisms thrive in near-marine salinities, such as those found in South SFB, and in the cooler temperatures of fall. Residence time could also influence the ability of AOA to bloom. In the PRE, high nitrification rates are correlated with longer residence times (65). South SFB is expected to have long residence times in summer and fall, on the order of months (66). Long residence times could allow populations to grow and consume excess ammonia from WWTPs. However, why NOB are not also favored during this time, especially with weeks to months of high nitrite concentrations, is unclear yet highly intriguing.

### SFB_3_bin18 has peak abundance in brackish salinities in North SFB.

SFB_3_bin18 has a peak abundance at station 3, recruiting 0.12% of metagenome reads and having a maximum relative abundance of 5.8 RPKG. ASV 1610 is identical to the 16S rRNA V4-V5 region of SFB_3_bin18 (Table S2). We observed a peak abundance of ASV 1610 in Suisun Bay (stations 3 and 6) in the late summer/early fall in salinities ranging from 8 to 12 PSU. Unlike in South Bay, where we saw high AOA relative abundance (>20%), ASV 1610 accounts for a maximum of only 0.2% of the 16S rRNA gene amplicon library (Fig. 1), occurring in September 2013. There is no correlation between ASV 1610 and nitrite concentration (*r^2^* = 0.0002, *P* > 0.05), and nitrite concentrations are not elevated as in South Bay (Fig. S2B). Intriguingly, in 2014 and 2015 we observed high concentrations of nitrite in the late summer and fall in Suisun Bay (Fig. S2B), which could be evidence for an AOA bloom or some other type of decoupling of ammonia and nitrite oxidation. However, since this nitrite accumulation occurs in the warmer temperatures of late summer and early fall and at mesohaline salinities, the underlying cause(s) may be distinct from what we observed in South SFB. A co-occurrence network identified 10 ASVs associated with ASV 1610, 6 of which had similar seasonal patterns in abundance to ASV 1610 and belonged to the NS4 marine group (*Flavobacteriaceae*), IS-44 (*Nitrosomonadaceae*), MB11C04 marine group (*Verrucomicrobiae*), *Alphaproteobacteria*, *Methylophilaceae*, and clade III (SAR11) (Fig. S4). The co-occurrence of these ASVs and ASV 1610 could indicate potential interactions or could be related to similar ecophysiologies. No NOB were associated with this AOA lineage, as has been observed for marine AOA and NOB ecotypes at different depths in Monterey Bay (67). Though the genus IS-44 falls into the *Nitrosomonadaceae*, it is unclear if organisms in this lineage are AOB.

### Similarity of SFB MAGs to other estuarine/coastal AOA.

**FIG 3:**
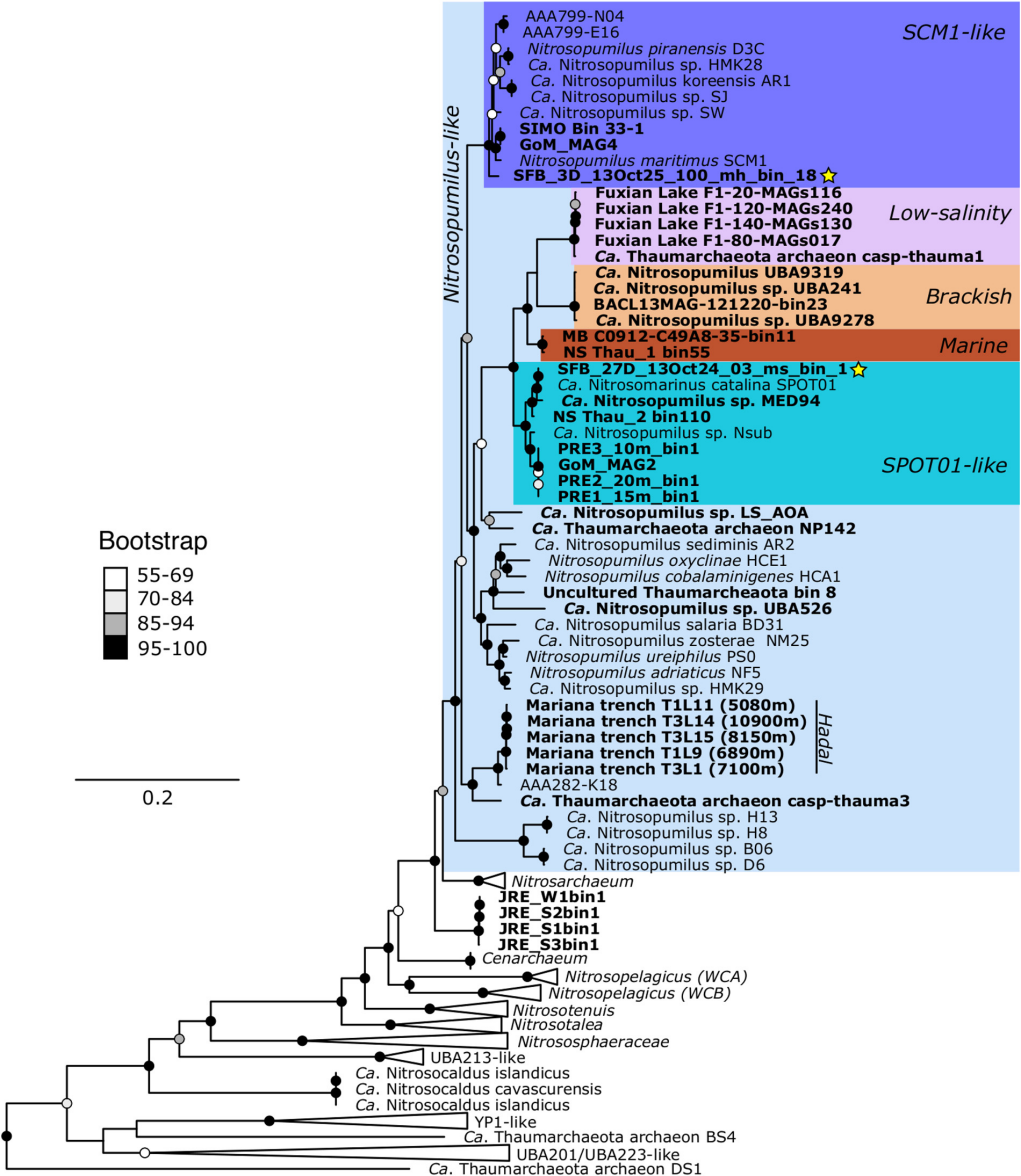
Concatenated ribosomal protein tree from MAFFT alignment of 24 ribosomal proteins from 153 genomes. The tree was constructed using IQtree and model LG+R5+F with 1,000 bootstraps. *Aigarchaeota* was used as an outgroup. MAGs are in bold, and MAGs generated in this study have a yellow star beside them. Background colors highlight subgroups of interest. Node color indicates bootstrap support.

We compared the genomic content of the representative MAGs generated in this study to medium- to high-quality (>70% complete, <10% contamination) AOA genomes from NCBI and IMG, along with additional MAGs from other pelagic estuary and coastal samples, including those from the: North Sea (68), Baltic Sea (69), PRE (64), JRE (65), Gulf of Mexico (GoM) (70), Sapelo Island (71), Amazon River (72, 73), and Monterey Bay (MB). Based on concatenated ribosomal trees, we identified two “pelagic” estuarine clusters within the *Nitrosopumilus* genus (Fig. 3). SFB_27_05_bin1 clusters with MAGs from the North Sea, Mediterranean Sea, PRE, JRE, and GoM (Fig. 3). SFB_3_bin18 clusters with MAGs from Sapelo Island, PRE, JRE, and GoM (Fig. 3). For simplicity, we refer to these two estuarine clusters as a “*Nitrosomarinus*-like” cluster and a *Nitrosopumilus* “SCM1-like” cluster based on well-known representative genomes from each cluster. The *amoA* phylogeny reveals the same clusters (Fig. S5).

10.1128/mSystems.01270-21.5 FIG S5 (A) Phylogeny of *amoA* based on a MAFFT alignment of 650 nucleotides. The tree was made using PhyML with the HKY85 substitution model and 50 bootstraps and is midpoint rooted. The MAGs generated in this study are in bold. (B) Phylogeny of *ureC* based on a MAFFT alignment of a 1,244-nucleotide sequence with all gaps manually removed. The tree was made using PhyML with the HKY85 substitution model and 50 bootstraps and is midpoint rooted. The MAG generated in this study is in bold. Download FIG S5, EPS file, 1.2 MB. Copyright © 2022 Rasmussen and Francis. 2022 Rasmussen and Francis. https://creativecommons.org/licenses/by/4.0/ This content is distributed under the terms of the Creative Commons Attribution 4.0 International license.

**FIG 4:**
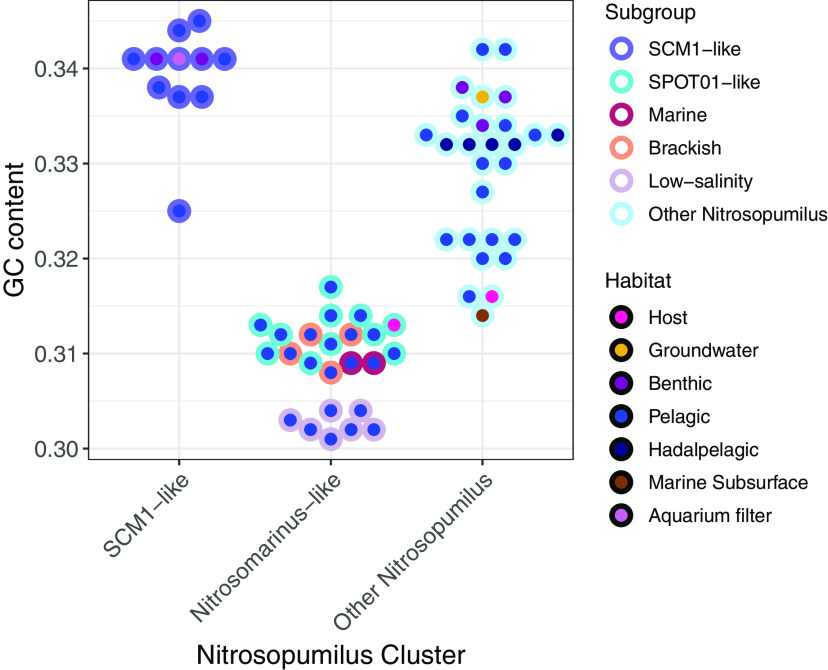
GC content for genomes in the SCM1-like, *Nitrosomarinus*-like, and other *Nitrosopumilus*-like clusters for genomes classified as *Nitrosopumilus* at the genus level using GTDB-tk and with >70% completeness and <5% contamination according to CheckM and GC content of >0.37 to remove higher-GC host-associated *Nitrosopumilus* genomes. Circle fill color indicates the habitat from which the genome originated, and circle border color indicates the subclade.

**FIG 5:**
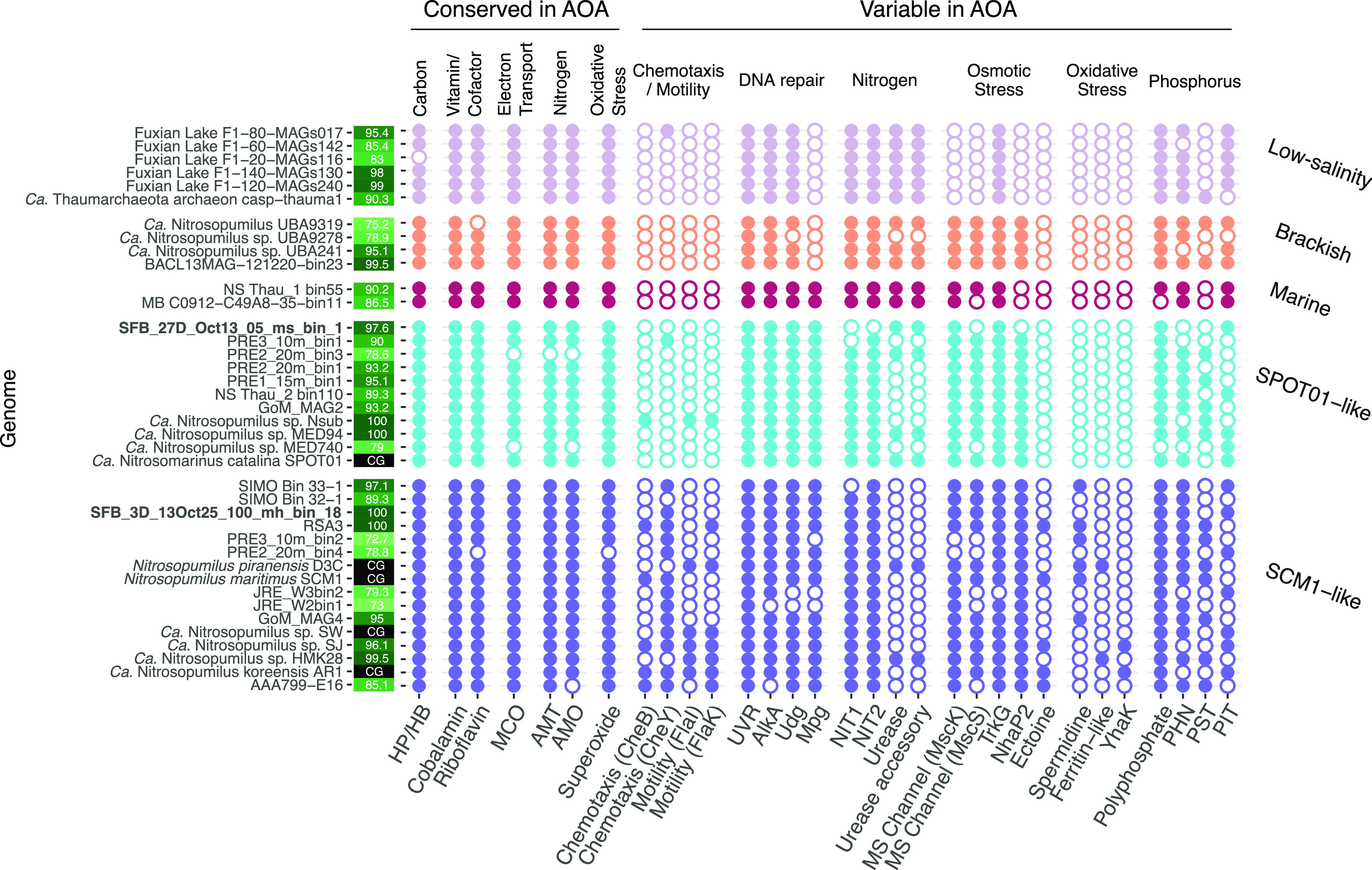
Gene presence or absence in pangenome based on COG, KO, or PFAM annotations. Solid circles represent gene presence, and open circles represent gene absence. Genome completeness is indicated in green squares next to genome names, while complete genomes are marked “CG” with a black background. MAGs generated in this study are in bold. Genes are clustered based on whether they are conserved or variable in AOA based on the work of Ren et al. (75) and Kerou et al. (108). Genes are further grouped by general function, labeled at the top of the plot. Genes displayed are as follows: HP/HB, Hcd (4-hydroxybutyryl-coenzyme A dehydratase); cobalamin, CobS (cobalamin synthase); riboflavin, RibB (3,4-dihydroxy-2-butanone 4-phosphate synthase); MCO, SufI (multicopper oxidase with three cupredoxin domains); AMT, AmtB (ammonia channel protein); AMO, *amoA* (ammonia monooxygenase subunit A); superoxide, SodA (superoxide dismutase); chemotaxis (CheB), CheB (chemotaxis response regulator); chemotaxis (CheY), CheY (chemotaxis response regulator receiver domain); motility (FlaI), archaeal flagellar protein; motility (FlaK), archaeal preflagellin peptidase; UVR, UvrA (UV-induced DNA lesion repair endonuclease ATPase subunit); AlkA, AlkA (DNA-3-methyladenine DNA glycosylase); Udg, Udg4/5 (uracil DNA glycosylase family 4/5); Mpg, 3-methyladenine DNA glycosylase gene; NIT1, YbeM (deaminated glutathione amidase); NIT2, YafV/Nit2 (nitrilase/omega-amidase); urease, UreC (urease alpha subunit); urease accessory, UreE (urease accessory protein); MS channel (MscK), small-conductance mechanosensitive channel; MS channel (MscS), small-conductance mechanosensitive channel; TrkG, TrkG (K^+^ transport system, membrane component); NhaP2, monovalent cation-proton antiporter; ectoine, EctD (ectoine hydroxylase); spermidine, SpeE (spermidine synthase); ferritin-like, Dps (DNA-binding ferritin-like protein); YhaK, YhaK (redox-sensitive bicupin protein); polyphosphate, Ppa (inorganic pyrophosphatase); PHN, PhnD (ABC-type phosphate/phosphonate transport system periplasmic component); PST, PstA (high-affinity ABC-type phosphate transport system permease component); PIT, PitA (low-affinity phosphate/sulfate permease).

We used a pangenomic workflow to compare a total of 39 AOA genomes, 23 and 16 from the *Nitrosomarinus*-like and SCM1-like clusters, respectively. This pangenome contained 5,140 gene clusters, of which 3,295 (64%) were annotated. In our comparison of genomes, we found that the *Nitrosomarinus-*like cluster had a relatively lower GC content (mean = 0.31) (Table 1) and smaller genome sizes (Fig. 4). In addition to this, we found that compared to the SCM1-like cluster, the *Nitrosomarinus*-like cluster was enriched in fewer genes (23 versus 44), possibly due to the smaller genome sizes. Most notably, a majority of *Nitrosomarinus*-like genomes contained urease and urease accessory genes, while most SCM1-like genomes did not (Fig. 5 and Fig. S6). While laboratory studies found that the SPOT01 strain could not grow on urea as a sole N source (41), urease may be of importance to other members of the clade. We also note that ectoine synthesis genes are missing in *Nitrosomarinus*-like organisms. Ectoine can function as an osmoprotectant in AOA (74) and the acquisition of ectoine synthesis genes may have been important for the expansion of AOA into the marine environment (75). Other genes enriched in *Nitrosomarinus*-like genomes include a sodium-proline symporter gene, *putP*, and a gene for an unknown transporter, *ynfA*.

**TABLE 1:** Summary statistics for the two estuarine AOA clusters (*Nitrosomarinus*-like and SCM1-like) in the pangenome

Pangenome cluster^*a*^	Avg GC content	Avg genome size (bp)	Avg no. of genes	Avg gene length (bp)	Avg no. of genes per kb	Avg no. of singletons
*Nitrosomarinus*-like	0.31	1,247,478	1,485	715	1.27	46.9
SCM1-like	0.34	1,559,476	1,762	740	1.23	77.0

a Includes only genomes that are >90% complete, which includes 21/39 genomes from the pangenome analysis.

10.1128/mSystems.01270-21.6 FIG S6 Genes that are enriched in (occurring significantly more frequently) *Nitrosomarinus*-like genomes (top facets) and SCM1-like (bottom facets). Filled circles indicate that the gene is present in the genome. The *x* axis is genome grouped by ecotype/subcluster. MAGs from SFB are in bold. Download FIG S6, EPS file, 1.0 MB. Copyright © 2022 Rasmussen and Francis. 2022 Rasmussen and Francis. https://creativecommons.org/licenses/by/4.0/ This content is distributed under the terms of the Creative Commons Attribution 4.0 International license.

Several coastal/estuarine genomes were classified as “*Nitrosopumilus catalinensis*” using GTDB (Table S3), and we refer to these as “SPOT01-like.” Organisms from this group can be transcriptionally and numerically dominant in meso- to euhaline estuarine and coastal sites (31, 38, 41). Organisms from eutrophic sites such as the GoM, JRE, and PRE cluster together (Fig. 3 and Fig. S7). PRE AOA genomes are enriched in heavy metal (e.g., manganese, zinc, and iron) transport genes as well as phosphate transport, indicating AOA are adapted to eutrophic conditions (64). Although PRE SPOT01-like MAGs were originally reported to not contain *ureC* (64), we recovered a *ureC* (along with all other urease subunits and accessory proteins) from PRE2_20m_bin3 (Fig. 5 and Fig. S5).

10.1128/mSystems.01270-21.7 FIG S7 Heat map based on ANI values for high-quality (>90% completeness and <5% contamination) genomes within the family *Nitrosopumilaceae* according to GTDB-tk. Download FIG S7, EPS file, 1.3 MB. Copyright © 2022 Rasmussen and Francis. 2022 Rasmussen and Francis. https://creativecommons.org/licenses/by/4.0/ This content is distributed under the terms of the Creative Commons Attribution 4.0 International license.

10.1128/mSystems.01270-21.10 TABLE S3 Metadata for estuarine and coastal MAGs analyzed for pangenomics and phylogenomics. Download Table S3, PDF file, 0.1 MB. Copyright © 2022 Rasmussen and Francis. 2022 Rasmussen and Francis. https://creativecommons.org/licenses/by/4.0/ This content is distributed under the terms of the Creative Commons Attribution 4.0 International license.

Within the *Nitrosomarinus*-like cluster, we found several clades of coastal/estuarine AOA found at marine salinities (referred to as “SPOT01-like” and “marine” in Fig. 3 and 5), a distinct cluster of organisms from the brackish waters of the Baltic Sea, and a cluster of organisms from freshwater to low-salinity sites (Fig. 3). The low-salinity organisms are missing genes related to osmoregulation (*mscK*, *mscS*, and *nhaP2*) (Fig. 5) and have particularly low GC content (Fig. 4). These salinity “ecotypes” within the *Nitrosomarinus*-like cluster appear to be under streamlining pressure, given the small genome sizes and apparent loss of genes to deal with osmoregulation. It is intriguing that these seemingly streamlined *Nitrosomarinus*-like organisms are dominant in estuarine and coastal waters, which are often nutrient rich, around the globe (38) and that they reach such high temporal abundances in SFB. While other marine AOA have streamlined genomes (41, 76), the exceptionally low GC content and high coding density point to potentially different selection pressures on *Nitrosomarinus*-like organisms.

Genomes in the SCM1-like cluster were enriched in many genes of unknown or predicted function compared to the *Nitrosomarinus*-like genomes (Fig. S6), possibly due to their larger size. SCM1-like genomes generally contained a *cheY* receiver domain (related to chemotaxis), spermidine synthase, and several putative amino acid symporters (*gltP* and *potE*) (Fig. S6), while *Nitrosomarinus*-like genomes did not. While they were not significantly enriched, it is worth noting that other genes related to motility and archaeal flagellin are more abundant in the SCM1-like cluster of genomes than in *Nitrosomarinus*-like genomes (Fig. 5), although the SCM1 strain itself lacks archaeal flagellin genes. The only *Nitrosomarinus*-like organism with motility genes is host-associated *Nitrosopumilus* sp. strain Nsub. The GC content was low (mean = 0.34) for SCM1-like organisms but higher than for the *Nitrosomarinus* cluster (mean = 0.31). In general, metrics such as gene length and gene coding density point to even more streamlining in *Nitrosomarinus-*like than SCM1-like genomes (Table 1). The SCM1-like clade includes MAGs from the GoM, PRE, JRE, and Sapelo Island, as well as our North SFB MAG (Fig. 3; Fig. S5 and Table S1). Sapelo Island is known for having AOA blooms annually in the warm summer months, concurrent with nitrite accumulation and high nitrification rates (37, 77), highlighting that bloom organisms can come from both of the “pelagic” AOA clusters analyzed in our study. As observed in other studies, the form of phosphate transporter does not strictly follow phylogeny (38), and we note that most genomes have genes for either the high-affinity PST (PstA, a high-affinity ABC-type phosphate transport system permease component) transporter or the low affinity PIT (PitA, a low-affinity phosphate/sulfate permease) transporter (Fig. 5). The ranges of ANI between genomes are similar within both the *Nitrosomarinus*-like clade (0.83 to 0.99) and SCM1-like clade (0.77 to 0.99), as is the median ANI for each clade (0.85 and 0.86, respectively). The median ANI between the *Nitrosomarinus*-like and SCM1-like clades is 0.79 (Fig. S7).

Ahlgren et al. (41) designated the SPOT01 organism as its own genus, *Nitrosomarinus*, based on its 16S rRNA gene, concatenated core conserved genes, *amoA*, and *ureC* gene phylogeny. Qin et al. (38) used the range of ANI and amino acid identity (AAI) within *Nitrosopumilus* to suggest that these organisms fall within the genus *Nitrosopumilus* and should not be distinguished as *Nitrosomarinus*. It is difficult to assign a strict ANI cutoff for delineating a genus, and several methods exist for trying to define genera based solely on genomic information (78, 79); however, an argument can be made for also considering the phylogenetic, physiological, metabolic, and ecological context of a group of organisms when describing a genus. Our study examining MAGs from a variety of pelagic environments found that *Nitrosomarinus*-like genomes form their own separate phylogenetic cluster based on ribosomal and key marker genes, as previously reported (38, 41), but also that they have a distinct ecological niche, occurring primarily in the pelagic environment, having low GC content and even more streamlined genomes than other *Nitrosopumilus* species. The *Nitrosomarinus*-like subclade is enriched in urease genes compared to the SCM1-like subclade but is also enriched in fewer genes than SCM1-like organisms. Additionally, genomes within the *Nitrosomarinus*-like clade form distinct subclades that can be found along the salinity gradient, can occur in eutrophic environments, and can bloom in some estuaries. Several studies have also noted the predominance of *Nitrosomarinus*-like organisms in coastal and estuarine sites (31, 38, 41, 64, 65). Physiological experiments with SPOT01 showing a lower optimum growth temperature could help explain the propensity of blooms in cooler months and a distinct niche for these organisms to thrive in. Increased efforts to enrich and isolate organisms from this group are necessary to establish their potentially distinct metabolisms (i.e., urea consumption) and physiology (i.e., cooler growth optimum). Defining a new genus generally requires multiple lines of evidence; however, *Nitrosomarinus*-like organisms may very well be different enough from other *Nitrosopumilus* organisms to merit this distinction.

Blooms of these AOA in South SFB has important implications for N cycling and ecosystem health. South Bay is characterized by high ammonia inputs from large WWTPs, but urea measurements are currently lacking in SFB. Urea could be an important source of ammonia for nitrification. While the SPOT01 strain could not grow on urea as a sole N source, further studies are required to see what role urea could play for the AOA population that blooms in SFB, represented by SFB_27_05_bin1, and other organisms in the *Nitrosomarinus*-like clade. Furthermore, what environmental conditions allow these AOA to form massive blooms while preventing NOB from following suit when nitrite concentrations are high in SFB for several weeks is a critical open question.

### Conclusions.

We recovered two high-quality AOA genomes from the waters of SFB, one of which corresponds to a bloom AOA population in South SFB associated with significant nitrite accumulation. These MAGs are related to other coastal/estuarine AOA MAGs falling into a lower-GC *Nitrosomarinus*-like cluster as well as a *Nitrosopumilus* SCM1-like cluster. SFB_3_bin18 likely represents a distinct species based on its GTDB classification, while SFB_27_05_bin1 is classified as “*Ca*. Nitrosomarinus catalina.” The conditions that allow the SFB_27_05_bin1 lineage of AOA to bloom require further investigation; temperature, oxygen, salinity, turbidity, residence time, urea concentrations, or competition with phytoplankton could all play a role in bloom formation. Furthermore, genomes within the *Nitrosomarinus*-like cluster originate from pelagic sites ranging from fully marine salinities to middle to low salinities, have low GC content and high coding density, often contain urease genes, form a distinct phylogenetic cluster, and are abundant primarily in coastal environments. These findings support the idea that *Nitrosomarinus*-like organisms have a niche distinct from that of other *Nitrosopumilus*-like organisms.

## MATERIALS AND METHODS

### Sample collection.

Microbial biomass was collected for DNA extraction approximately monthly between April 2012 and March 2014 during United States Geological Survey (USGS) water quality monitoring cruises in the channel of the SFB estuary onboard the R/V *Polaris*. Microbial cells were collected from surface waters (2 m) and bottom waters (1 m above the estuary floor) by pressure filtering 150 to 1,000 mL of water from conductivity, temperature, and depth (CTD) instrument package casts through a 10-μm-pore-size polycarbonate Isopore membrane filter (47-mm diameter; EMD Millipore, Darmstadt, Germany) in line with a 0.22-μm polyethersulfone Supor-200 membrane filter (47-mm diameter; Pall, Port Washington, NY), followed by flash freezing of 0.22-μm filters in liquid nitrogen prior to storage at −80°C.

### Environmental data.

Corresponding water quality data from the exact sampling cruises, stations, and water depths used in this study were downloaded from the USGS Water Quality of SFB database (80). Additional ammonium, nitrate, and nitrite measurements were performed on water samples using filtered (0.22 μm pore size) water that was frozen on dry ice prior to storage at −20°C. Ammonium was measured using the salicylate-hypochlorite method (81). Nitrate and nitrite were measured using a SmartChem200 discrete analyzer (Unity Scientific, Brookfield, CT) following standard manufacturer’s operating procedures. Nutrients were measured within 1 week of sample collection.

### DNA extraction.

DNA was extracted with the FastDNA spin kit for soil (MP Biomedicals, Santa Ana, CA), following the manufacturer’s instructions, with the following modifications: filters were homogenized in bead tubes for 40 s at speed 6.0 in a FastPrep bead beater (MP Biomedicals, Santa Ana, CA), and final DNA was eluted in 75 μl of 55°C sterile DNase-free water. DNA was quantified using the Qubit double-stranded DNA (dsDNA) broad-range assay (Life Technologies, Grand Island, NY) and stored at −80°C.

### 16S rRNA gene amplicon sequencing and processing.

16S rRNA gene amplicon library preparation and sequencing were conducted through a Department of Energy Joint Genome Institute (JGI) Community Science Program (CSP) project and through the Georgia Genomics and Bioinformatics Core (GGBC) at the University of Georgia. In total, 177 bottom water samples and 20 surface samples were amplified with the 16S V4-V5 primers (82) (515F-Y, GTGYCAGCMGCCGCGGTAA; 926R, CCGYCAATTYMTTTRAGTTT). Library preparation was performed by JGI and GGBC following their standard operating procedures. The JGI protocol is available at https://jgi.doe.gov/user-programs/pmo-overview/protocols-sample-preparation-information/. GGBC uses an annealing temperature of 66°C and follows the Illumina sample preparation guide: https://support.illumina.com/documents/documentation/chemistry_documentation/16s/16s-metagenomic-library-prep-guide-15044223-b.pdf. Samples were pooled and sequenced on an Illumina MiSeq instrument.

Cutadapt (v3.1) (83) was used to remove primer sequences from amplicons. The DADA2 (84) pipeline was used to create amplicon sequence variants (ASVs) following the standard pipeline available at https://benjjneb.github.io/dada2/tutorial.html. Briefly, reads were trimmed and filtered prior to learning error rates for each plate separately. Inferred sequence variants were then created, forward and reverse reads were merged, and sequence variants were dereplicated. The four sequencing plates were then merged into a single sequence table, and chimeras were removed. Taxonomy was assigned using Silva SSU r138 with the DECIPHER Bioconductor package (85). Libraries with fewer than 10,000 sequences were removed from analysis, and ASVs with fewer than 4 reads in 3 samples were removed.

### Co-occurrence networks.

Co-occurrence networks were made for station 27 bottom water samples and for station 6 samples to investigate potential associations between the AOA and other community members. ASVs were filtered to have at least 50 reads in at least 3 samples to remove low-abundance ASVs that might cause spurious associations. Networks were made using SPIEC-EASI (sparse inverse covariance estimation for ecological association and statistical inference) (86) following standard practices using the neighborhood selection (MB) model. Igraph objects were made based on StARS-refit network. Subgraphs with nodes sharing direct edges with ASV 8 and ASV 1610 were selected using Cytoscape (87).

### Quantitative PCR analysis of marine group I *Thaumarchaeota* 16S rRNA gene abundance.

qPCR was performed on microbial DNA samples from station 27 using a fluorescent TaqMan assay. The assay amplified a region of the 16S rRNA gene of marine group I (MGI) *Thaumarchaeota* (representing AOA abundance) (88), using primers GI_751F (GTCTACCAGAACAYGTTC) and GI_956R (HGGCGTTGACTCCAATTG) and TaqMan probe MGI_889 FAM-BHQ (5′-6-carboxyfluorescein [FAM]-AGTACGTGAA-BHQ1a-Q-3′) (89). Samples and standards were run in triplicate. Reaction mixtures contained a total volume of 25 μl, including 1 μl of template DNA, 0.5 μl of primer (10 μM), 0.75 μl of probe (10 μM), 10.75 μl of TaqMan environmental master mix 2.0 (Applied Biosystems), and 11.5 μl of double-distilled water (ddH_2_O) and was run on an ABI StepOnePlus real-time PCR system (Applied Biosystems) as follows: 95°C for 10 min followed by 40 cycles of 95°C for 15 s and 55°C for 1 min. Standards were prepared by a dilution series of 10^−1^ to 10^−7^ copies per reaction of linearized plasmids. All standard curves had *r^2^* values of ≥0.99.

### Metagenome sequencing, assembly, and binning.

Metagenomes from October 2013 were sequenced via JGI CSP (proposal ID 503022) on an Illumina HiSeq 2500-1TB instrument. Quality-controlled filtered raw metagenome data (JGI Project IDs 1167699 to 1167707) were downloaded from JGI Genome Portal for assembly, binning, and refining using the metaWRAP (v1.3.2) pipeline (90). Metagenomes were assembled using MEGAHIT (v1.1.3) (91) following default parameters and a range of kmers (*k* = 21, 29, 39, 59, 79, 99, 119, 141). Metagenomes were subset using seqtk (seed -s100) to allow targeted assembly of highly abundant AOA. Additional assemblies were made using both MEGAHIT and metaSPAdes (v3.13.0) (92) using 1%, 3%, 5%, 10%, and 20% of metagenome reads. Assemblies were then binned using contigs of >2,000 nucleotides (nt) using both MetaBAT2 (v2.12.1) (93) and MaxBin 2.0 (v2.2.6) (94). Coassemblies of stations based on salinity zone were also done to target lower-abundance nitrifiers and binned using contigs of >2,500 nt using MetaBAT2 and MaxBin 2.0 plus CONCOCT (v1.1.0) (95) for coassemblies. Bins were consolidated and filtered using metawrap bin_refinement to be >50% complete and have <10% contamination. These consolidated bins were then reassembled with metaSPAdes using strict or permissive algorithm using metawrap reassemble_bins. Completeness and contamination of MAGs were calculated using CheckM (v1.1.3) (96). Reads were competitively recruited to MAGs using Bowtie2 (v2.4.2) (97) and the default parameters. Abundances are displayed as unpaired reads recruited per genome size of MAG in kilobases of MAG divided by gigabase of metagenome (RPKG). Genes were called using Prodigal (v2.6.3) (98). Translated sequences from each AOA MAG were annotated using the RAST tool kit (RAST-tk v2.0) (99, 100) for SEED (101) annotation and GhostKOALA (102) for KEGG KO annotations. Taxonomic classification for each MAG was performed using the Genome Taxonomy Database toolkit (GTDB-tk) (103) with the database release 05-RS95. MAGs were dereplicated using dRep (v2.3.2) (104) at 98% ANI.

### Phylogenomic and pangenomic analysis.

All *Nitrososphaerales* genomes in the GTDB database (release 05-RS95, 17 July 2020) were downloaded from the National Center of Biotechnology Information (NCBI) database (*n* = 170), and all genomes annotated as *Thaumarchaeota* that were nonredundant with the NCBI data set were downloaded from the Integrated Microbial Genome (IMG) database (*n* = 177) in January 2021. Additional pelagic estuarine and coastal *Thaumarchaeota* MAGs were downloaded from several other sequence databases (see Table S1 for full details on database, study citations, and accession numbers). anvi’o (hope v7) (105) was used to annotate, analyze, and compare all of the downloaded *Thaumarchaeota* genomes, in addition to the two finalized AOA MAGs generated in this study, following the standard phylogenomics and pangenomics workflows. Contigs shorter than 500 bp were excluded from the analysis. Conserved ribosomal and housekeeping genes were annotated with the 7 default hmm databases used by anvi-run-hmms with the argument –also-scan-trnas, which uses tRNAscan-SE (v2.0.7) to annotate tRNAs. All genomes were annotated through the anvi’o pipeline using 3 databases, including COG (anvi-run-ncbi-cogs, Diamond set to fast), KEGG (anvi-run-kegg-kofams, using hmmsearch), and Pfam (anvi-run-pfams, using hmmsearch). Completion and contamination were calculated using CheckM (v 1.1.3). Concatenated and aligned amino acid sequences were retrieved from MAGs using anvi-get-sequences-for-hmm-hits for all genomes with <5% contamination and >70% complete and containing 30 of 36 archaeal ribosomal genes, leaving 153 genomes in the analysis. Genes not occurring in at least 145 of 153 genomes were removed, yielding 24 remaining ribosomal genes, including L16, S8, S3Ae, L29, L26, S9, S17, S11, S2, L6, S13, L32e, S8e, L3, L4, L13, S12_S23, S19, L14, L22, L21e, L23, L1, and S7. Trimal was used to remove gaps in amino acid alignments with less than 80% coverage. Then, IQ-TREE -m MFP was used for extended model selection for phylogeny of MAGs. IQ-TREE was used to construct the final phylogenomic tree using model LG+R5+F with 1,000 bootstraps. Based on clusters in the tree and the GTDB species annotations, a pangenomics analysis was performed to compare two different groups within the *Nitrosopumilaceae*. Genomes with >10% contamination and <70% completeness were removed from pangenomics workflow, leading to a total of 39 genomes included in the pangenome. The ANI of genomes was calculated using pyANI (106) through anvi’o using default parameters. Gene enrichment between groups was calculated using the anvi-compute-functional-enrichment function for all three annotation sources, and significant genes were selected based on the adjusted *P* value to correct for multiple testing. Gene sequences of interest (i.e., *amoA* and *ureC*) were collected from pangenomes using anvi-get-sequences-for-gene-clusters.

### Statistical analysis.

Regressions were made using Pearson correlation with the cor() function from base R (107) stats package.

### Data availability.

Amplicon libraries of 16S rRNA genes are available in the NCBI SRA under BioProject no. PRJNA577706. Metagenomes are available under NCBI BioProject no. PRJNA439806 through PRJNA439813. The final set of two AOA MAGs were deposited in NCBI under Biosample no. SAMN22441885 and SAMN22441886. SFB water quality data are available from the USGS database: https://sfbay.wr.usgs.gov/water-quality-database/.
