# Supplementary material for: Genome-Resolved Metagenomic Insights into Massive Seasonal Ammonia-Oxidizing Archaea Blooms in San Francisco Bay
Source: mSystems. 2022 Jan 25;7(1):e01270-21. doi: 10.1128/msystems.01270-21 (PMC8788347; doi:10.1128/msystems.01270-21)
Supplement: TABLE S1 [file msystems.01270-21-st001.pdf]

**Table S1** Ammonia-oxidizing archaea bin metadata

| Bin Name | Complet-ness (%) | Contam-ination (%) | GC | N50 | Size (bp) | Cluster | Dereplicated representative |
|---|---|---|---|---|---|---|---|
| SFB_3D_13Oct25_100_mh_bin_18_orig | 100 | 0.97 | 0.341 | 36644 | 1343525 | SCM1-like | yes |
| SFB_Oligo_13Oct25_mh_bin_43 | 98.05 | 0.00 | 0.34 | 27682 | 1321591 | SCM1-like | |
| SFB_3D_13Oct25_50_mh_bin_29_permissive | 90.29 | 0.00 | 0.338 | 10285 | 1308268 | SCM1-like | |
| SFB_27D_13Oct24_20_ms_bin_9_orig | 96.6 | 1.94 | 0.316 | 19749 | 1336932 | Nitrosomarinus-like | |
| SFB_27D_13Oct24_10_ms_bin_2_orig | 97.08 | 0.49 | 0.314 | 19882 | 1271503 | Nitrosomarinus-like | |
| SFB_27D_13Oct24_05_ms_bin_1_permissive | 97.57 | 0.00 | 0.313 | 40964 | 1218214 | Nitrosomarinus-like | yes |
| SFB_34D_13Oct24_10_ms_bin_4_strict | 99.02 | 2.91 | 0.312 | 29431 | 1200679 | Nitrosomarinus-like | |
| SFB_27D_13Oct24_03_ms_bin_1_permissive | 99.02 | 0.00 | 0.314 | 37857 | 1180840 | Nitrosomarinus-like | |
| SFB_34D_13Oct24_20_ms_bin_10_orig | 96.6 | 0.97 | 0.313 | 19489 | 1164262 | Nitrosomarinus-like | |
| SFB_27D_13Oct24_03_mh_bin_1_strict | 87.86 | 2.54 | 0.314 | 10599 | 1133121 | Nitrosomarinus-like | |
| SFB_34D_13Oct24_05_ms_bin_1_strict | 93.68 | 0.00 | 0.313 | 24421 | 1116413 | Nitrosomarinus-like | |
| SFB_18D_13Oct24_20_ms_bin_3_oirg | 93.68 | 0.97 | 0.313 | 7557 | 1070533 | Nitrosomarinus-like | |
| SFB_27D_13Oct24_01_ms_bin_1_permissive | 96.11 | 0.97 | 0.313 | 9545 | 1050185 | Nitrosomarinus-like | |
| SFB_27D_13Oct24_01_mh_bin_1_permissive | 89.24 | 0.97 | 0.314 | 11865 | 1015501 | Nitrosomarinus-like | |
| SFB_34D_13Oct24_03_ms_bin_1_permissive | 86.81 | 0.24 | 0.314 | 9468 | 996541 | Nitrosomarinus-like | |
| SFB_34D_13Oct24_03_mh_bin_1_permissive | 82.52 | 0.24 | 0.313 | 8492 | 973454 | Nitrosomarinus-like | |
| SFB_13D_13Oct25_20_ms_bin_7_orig.fa | 87.13 | 0.32 | 0.313 | 5724 | 966060 | Nitrosomarinus-like | |
| SFB_27D_13Oct24_05_mh_bin_1_strict | 75.32 | 0.00 | 0.313 | 14070 | 934250 | Nitrosomarinus-like | |
| SFB_34D_13Oct24_05_mh_bin_1_permissive | 87.21 | 1.94 | 0.315 | 13410 | 928062 | Nitrosomarinus-like | |
| SFB_18D_13Oct24_50_mh_bin_10_permissive | 77.34 | 3.24 | 0.307 | 3988 | 789838 | Nitrosomarinus-like | |
