# Supplementary material for: Genome-Resolved Metagenomic Insights into Massive Seasonal Ammonia-Oxidizing Archaea Blooms in San Francisco Bay
Source: mSystems. 2022 Jan 25;7(1):e01270-21. doi: 10.1128/msystems.01270-21 (PMC8788347; doi:10.1128/msystems.01270-21)
Supplement: TABLE S2 [file msystems.01270-21-st002.pdf]

Table S2 Similarity of 16S rRNA V4-V5 regions from representative AOA MAGs and top 20 *Thaumarchaeota* ASVs in 16S rRNA gene amplicons

ASV/MAG 16S rRNA V4-V5 region	8	SFB_27_05_bin1	754	1111	463	638	1347	1610	SFB_3_bin18	1657	485	622	1467	1345	1193	522	1433	950	2193	979	1892	1181
**8**		100	99.735	99.735	99.469	99.469	99.735	99.735	99.735	99.735	99.469	98.408	98.408	98.674	98.939	97.613	97.347	97.347	96.817	97.613	96.286	94.695
**SFB_27_05_bin1**	100		99.735	99.735	99.469	99.469	99.735	99.735	99.735	99.735	99.469	98.408	98.408	98.674	98.939	97.613	97.347	97.347	96.817	97.613	96.286	94.695
**754**	99.735	99.735		99.469	99.204	99.204	99.469	99.469	99.469	99.469	99.204	98.674	98.143	98.939	98.674	97.347	97.082	97.082	96.552	97.347	96.552	94.43
**1111**	99.735	99.735	99.469		99.204	99.204	99.469	99.469	99.469	99.469	99.204	98.143	98.143	98.408	99.204	97.347	97.082	97.082	96.552	97.347	96.552	94.96
**463**	99.469	99.469	99.204	99.204		98.939	99.204	99.204	99.204	99.204	98.939	97.878	97.878	98.143	98.408	97.082	96.817	96.817	96.286	97.082	95.756	94.164
**638**	99.469	99.469	99.204	99.204	98.939		99.204	99.204	99.204	99.204	98.939	97.878	97.878	98.143	98.408	97.082	97.347	96.817	96.286	97.082	95.756	94.164
**1347**	99.735	99.735	99.469	99.469	99.204	99.204		99.469	99.469	99.469	99.204	98.143	98.143	98.408	98.674	97.347	97.082	97.082	96.552	97.347	96.021	94.43
**1610**	99.735	99.735	99.469	99.469	99.204	99.204	99.469		100	99.469	99.204	98.143	98.143	98.408	98.674	97.347	97.082	97.613	97.082	97.347	96.021	94.43
**SFB_3_bin18**	99.735	99.735	99.469	99.469	99.204	99.204	99.469	100		99.469	99.204	98.143	98.143	98.408	98.674	97.347	97.082	97.613	97.082	97.347	96.021	94.43
**1657**	99.735	99.735	99.469	99.469	99.204	99.204	99.469	99.469	99.469		99.204	98.143	98.143	98.408	98.674	97.347	97.082	97.082	96.552	97.347	96.021	94.43
**485**	99.469	99.469	99.204	99.204	98.939	98.939	99.204	99.204	99.204	99.204		97.878	97.878	98.143	98.408	97.082	96.817	96.817	96.286	97.082	95.756	94.164
**622**	98.408	98.408	98.674	98.143	97.878	97.878	98.143	98.143	98.143	98.143	97.878		99.469	99.204	98.939	96.021	95.756	95.756	95.225	96.021	95.225	93.103
**1467**	98.408	98.408	98.143	98.143	97.878	97.878	98.143	98.143	98.143	98.143	97.878	99.469		99.204	98.939	96.552	96.286	96.286	95.756	96.552	94.96	93.103
**1345**	98.674	98.674	98.939	98.408	98.143	98.143	98.408	98.408	98.408	98.408	98.143	99.204	99.204		99.204	96.817	96.552	96.552	96.021	96.817	95.756	93.369
**1193**	98.939	98.939	98.674	99.204	98.408	98.408	98.674	98.674	98.674	98.674	98.408	98.939	98.939	99.204		96.552	96.286	96.286	95.756	96.552	95.756	94.164
**522**	97.613	97.613	97.347	97.347	97.082	97.082	97.347	97.347	97.347	97.347	97.082	96.021	96.552	96.817	96.552		99.735	99.735	99.204	99.469	97.082	94.43
**1433**	97.347	97.347	97.082	97.082	96.817	97.347	97.082	97.082	97.082	97.082	96.817	95.756	96.286	96.552	96.286	99.735		99.469	98.939	99.204	96.817	94.164
**950**	97.347	97.347	97.082	97.082	96.817	96.817	97.082	97.613	97.613	97.082	96.817	95.756	96.286	96.552	96.286	99.735	99.469		99.469	99.204	96.817	94.164
**2193**	96.817	96.817	96.552	96.552	96.286	96.286	96.552	97.082	97.082	96.552	96.286	95.225	95.756	96.021	95.756	99.204	98.939	99.469		98.674	96.286	94.695
**979**	97.613	97.613	97.347	97.347	97.082	97.082	97.347	97.347	97.347	97.347	97.082	96.021	96.552	96.817	96.552	99.469	99.204	99.204	98.674		97.347	94.43
**1892**	96.286	96.286	96.552	96.552	95.756	95.756	96.021	96.021	96.021	96.021	95.756	95.225	94.96	95.756	95.756	97.082	96.817	96.817	96.286	97.347		94.43
**1181**	94.695	94.695	94.43	94.96	94.164	94.164	94.43	94.43	94.43	94.43	94.164	93.103	93.103	93.369	94.164	94.43	94.164	94.164	94.695	94.43	94.43	
