# Supplementary material for: Genome-Resolved Metagenomic Insights into Massive Seasonal Ammonia-Oxidizing Archaea Blooms in San Francisco Bay
Source: mSystems. 2022 Jan 25;7(1):e01270-21. doi: 10.1128/msystems.01270-21 (PMC8788347; doi:10.1128/msystems.01270-21)
Supplement: TABLE S3 [file msystems.01270-21-st003.pdf]

Table S3 Metadata for estuarine and coastal MAGs analyzed for pangenomics and phylogenomics

Name in this study	Original name	Study	Study site	Data-base	Accession ID	Estuarine/ Coastal	Habitat type	GTDB Genus	GTDB species
AR_AM0615_GCA_900299125_1	AM_0615	Santos-Junior et al., 2017	Amazon River Upstream section	NCBI	GCA_900299125.1	Y	Pelagic	Nitrosotenuis	NA
AR_ThauR71_2778260922	ThauR71	Pinto et al., 2020	Amazon River Plume	IMG	2778260922	Y	Pelagic	Nitrosotenuis	Nitrosotenuis sp002499525
ARP_ThauP25_2781125712	ThauP25	Pinto et al., 2020	Amazon River Plume	IMG	2781125713	Y	Pelagic	Nitrosotenuis	Nitrosotenuis sp002499525
ARP_ThauP41_2781125713	ThauP41	Pinto et al., 2020	Amazon River Plume	IMG	2781125712	Y	Pelagic	Nitrosopelagicus	NA
BS_BACL13_120910_bin56_GCA_001438895_1	BACL13 MAG-120910-bin56	Hugerth et al., 2015	Baltic Sea	NCBI	GCA_001438895.1	Y	Pelagic	Nitrosopumilus	Nitrosopumilus sp001437625
BS_BACL13_121220_bin23_GCA_001437625_1	BACL13 MAG-121220-bin23	Hugerth et al., 2015	Baltic Sea	NCBI	GCA_001437625.1	Y	Pelagic	Nitrosopumilus	Nitrosopumilus sp001437625
GoM_MAG1_GCA_003702485_1	GoM_MAG1	Georgia Institute of Technology	Gulf of Mexico	NCBI	GCA_003702485.1	Y	Pelagic	NA	NA
GoM_MAG2_GCA_003702465_1	GoM_MAG2	Georgia Institute of Technology	Gulf of Mexico	NCBI	GCA_003702465.1	Y	Pelagic	Nitrosopumilus	Nitrosopumilus sp003702465
GoM_MAG3_GCA_003702585_1	GoM_MAG3	Georgia Institute of Technology	Gulf of Mexico	NCBI	GCA_003702585.1	Y	Pelagic	NA	NA
GoM_MAG4_GCA_003702495_1	GoM_MAG4	Georgia Institute of Technology	Gulf of Mexico	NCBI	GCA_003702495.1	Y	Pelagic	Nitrosopumilus	Nitrosopumilus sp003702495
GoM_MAG5_GCA_003702525_1	GoM_MAG5	Georgia Institute of Technology	Gulf of Mexico	NCBI	GCA_003702525.1	Y	Pelagic	NA	NA
GoM_MAG6_GCA_003702545_1	GoM_MAG6	Georgia Institute of Technology	Gulf of Mexico	NCBI	GCA_003702545.1	Y	Pelagic	NA	NA
JRE_S1bin1	S1bin1	Zou et al., 2020	Jiulong River Estuary	NODE	OER057304	Y	Benthic	NA	NA
JRE_S2bin1	S2bin1	Zou et al., 2020	Jiulong River Estuary	NODE	OER057305	Y	Benthic	NA	NA
JRE_S3bin1	S3bin1	Zou et al., 2020	Jiulong River Estuary	NODE	OER057306	Y	Benthic	NA	NA
JRE_W1bin1	W1bin1	Zou et al., 2020	Jiulong River Estuary	NODE	OER057298	Y	Pelagic	NA	NA
JRE_W2bin1	W2bin1	Zou et al., 2020	Jiulong River Estuary	NODE	OER057299	Y	Pelagic	Nitrosopumilus	Nitrosopumilus sp003702495
JRE_W2bin2	W2bin2	Zou et al., 2020	Jiulong River Estuary	NODE	OER057300	Y	Pelagic	Nitrosopumilus	NA
JRE_W2bin3	W2bin3	Zou et al., 2020	Jiulong River Estuary	NODE	OER057301	Y	Pelagic	NA	NA
JRE_W3bin1	W3bin1	Zou et al., 2020	Jiulong River Estuary	NODE	OER057302	Y	Pelagic	Nitrosopumilus	NA
JRE_W3bin2	W3bin2	Zou et al., 2020	Jiulong River Estuary	NODE	OER057303	Y	Pelagic	Nitrosopumilus	NA
NS_Thau1_20110321_Bin_55_1	Thau_1	Orellana et al., 2019	North Sea	ENA	SAMEA5404198	Y	Pelagic	Nitrosopumilus	NA
NS_Thau2_20100303_Bin_110_1	Thau_2	Orellana et al., 2019	North Sea	ENA	SAMEA5403578	Y	Pelagic	Nitrosopumilus	Nitrosopumilus catalinensis
PRE1_15m_bin1	PRE1_15m_bin1	Zou et al., 2019	Pearl River Estuary	NCBI	SRR10579427	Y	Pelagic	Nitrosopumilus	Nitrosopumilus sp003702465
PRE1_25m_bin3	PRE1_25m_bin3	Zou et al., 2019	Pearl River Estuary	NCBI	SRR10579426	Y	Pelagic	Nitrosopelagicus	NA

PRE2_20m_bin1	PRE2_20m_bin 1	Zou et al., 2019	Pearl River Estuary	NCBI	SRR10579425	Y	Pelagic	Nitrosopumilus	Nitrosopumilus sp003702465
PRE2_20m_bin3	PRE2_20m_bin 3	Zou et al., 2019	Pearl River Estuary	NCBI	SRR10579424	Y	Pelagic	Nitrosopumilus	Nitrosopumilus sp003702465
PRE2_20m_bin4	PRE2_20m_bin 4	Zou et al., 2019	Pearl River Estuary	NCBI	SRR10579423	Y	Pelagic	Nitrosopumilus	NA
PRE3_10m_bin1	PRE3_10m_bin 1	Zou et al., 2019	Pearl River Estuary	NCBI	SRR10579422	Y	Pelagic	Nitrosopumilus	Nitrosopumilus sp003702465
PRE3_10m_bin2	PRE3_10m_bin 2	Zou et al., 2019	Pearl River Estuary	NCBI	SRR10579421	Y	Pelagic	Nitrosopumilus	Nitrosopumilus sp003702495
SFB_27D_13Oct24_05_ms_bin_1	SFB_27_05_bin 1	This study	San Francisco Bay	NCBI	PRJNA439812	Y	Pelagic	Nitrosopumilus	Nitrosopumilus catalinensis
SFB_3D_13Oct25_100_mh_bin_18	SFB_3_bin18	This study	San Francisco Bay	NCBI	PRJNA439812	Y	Pelagic	Nitrosopumilus	NA
SI_GCA_008080815	SIMO Bin 32-1	Damashek et al., 2019	Sapelo Island, Georgia	NCBI	GCA_008080815.1	Y	Pelagic	Nitrosopumilus	NA
SI_GCA_008080855	SIMO Bin 33-1	Damashek et al., 2019	Sapelo Island, Georgia	NCBI	GCA_008080855.1	Y	Pelagic	Nitrosopumilus	Nitrosopumilus sp003702495
Ogasawara_trench_C4_9697m	Ogasawara trench C4 (9697m)	Wang et al., 2019	Hadal_waters	BIGD	SAMC026836	N	Pelagic	Nitrosopumilus	Nitrosopumilus sp007036605
Mariana_trench_T1L11_5080m	Mariana trench T1L11 (5080m)	Wang et al., 2019	Hadal_waters	BIGD	SAMC021086	N	Pelagic	Nitrosopumilus	Nitrosopumilus sp007036605
Mariana_trench_T1L9_6890m	Mariana trench T1L9 (6890m)	Wang et al., 2019	Hadal_waters	BIGD	SAMC021091	N	Pelagic	Nitrosopumilus	Nitrosopumilus sp007036605
Mariana_trench_T3L1_7100m	Mariana trench T3L1 (7100m)	Wang et al., 2019	Hadal_waters	BIGD	SAMC021092	N	Pelagic	Nitrosopumilus	Nitrosopumilus sp007036605
Mariana_trench_T3L14_10900m	Mariana trench T3L14 (10900m)	Wang et al., 2019	Hadal_waters	BIGD	SAMC021095	N	Pelagic	Nitrosopumilus	Nitrosopumilus sp007036605
Mariana_trench_T3L15_8150m	Mariana trench T3L15 (8150m)	Wang et al., 2019	Hadal_waters	BIGD	SAMC021093	N	Pelagic	Nitrosopumilus	Nitrosopumilus sp007036605
Mariana_trench_T3L19_10890m	Mariana trench T3L19 (10890m)	Wang et al., 2019	Hadal_waters	BIGD	SAMC021096	N	Pelagic	Nitrosopelagicus	Nitrosopelagicus sp000484935
Mariana_trench_D17_5900m	Mariana trench D17 (5900m)	Wang et al., 2019	Hadal_waters	BIGD	SAMC021089	N	Pelagic	Nitrosopumilus	Nitrosopumilus sp007036605
Mariana_trench_D1_5400m	Mariana trench D1 (5400m)	Wang et al., 2019	Hadal_waters	BIGD	SAMC021088	N	Pelagic	Nitrosopelagicus	Nitrosopelagicus sp007037745
Ogasawara_trench_F20_2015m	Ogasawara trench F20 (2015m)	Wang et al., 2019	Hadal_waters	BIGD	SAMC026834	N	Pelagic	NA	NA
GCA_013407385_1	Ca. Nitrosarchaeum limnosediminis AC2	Qin et al., 2019	Near-shore sediments of Lake Acton, USA	NCBI	GCA_013407385.1	N	Benthic	Nitrosarchaeum	Nitrosarchaeum koreense
GCA_013407275_1	Ca. Nitrosotenuis limnosediminis DW1	Qin et al., 2019	Near-shore sediments of Lake Delaware, USA	NCBI	GCA_013407275.1	N	Benthic	Nitrosotenuis	NA
GCA_013407145_1	Nitrosopumilus cobalaminigene s HCA1	Qin et al., 2019	50m depth coastal seawater from Hood Canal, Washington	NCBI	GCA_013407145.1	Y	Pelagic	Nitrosopumilus	NA

GCA_013407165_1	Nitrosopumilus oxyclinae HCE1	Qin et al., 2019	17m depth coastal seawater from Hood Canal, Washington	NCBI	GCA_013407165.1	Y	Pelagic	Nitrosopumilus	NA
GCA_014078545_1	Ca. Cenarchaeum sp. HMK20	Qin et al., 2019	10 m depth coastal seawater at Lynch Cove, Washington	NCBI	GCA_014078545.1	Y	Pelagic	Cenarchaeum	NA
GCA_014078535_1	Ca. Nitrosopumilus sp. HMK28	Qin et al., 2019	Bonita beach seawater, Florida	NCBI	GCA_014078535.1	Y	Pelagic	Nitrosopumilus	Nitrosopumilus piranensis
GCA_014078525_1	Ca. Nitrosopumilus sp. HMK29	Qin et al., 2019	Pine Island seawater, Florida	NCBI	GCA_014078525.1	Y	Pelagic	Nitrosopumilus	NA
GCA_003175215_1	Ca. Nitrosopumilus zosterae NM25	Qin et al., 2019	0–5 cm below the eelgrass sediments	DDBJ	PRJDB6801	Y	Benthic	Nitrosopumilus	Nitrosopumilus sp003175215
GCA_013407185_1	Nitrosopumilus ureiphilus PS0	Qin et al., 2019	Nearshore marine surface sediment, Washington	NCBI	GCA_013407185.1	Y	Benthic	Nitrosopumilus	NA
MB_C0912_C49A8_35_bin11	C0912_C49A8_35_bin11	Stanford University	Monterey Bay	IMG	3300027298_11	Y	Pelagic	Nitrosopumilus	NA
